# Voltage gated sodium channels in cancer and their potential mechanisms of action

**DOI:** 10.1080/19336950.2019.1666455

**Published:** 2019-09-24

**Authors:** Madeline Angus, Peter Ruben

**Affiliations:** Department of Biomedical Physiology and Kinesiology, Simon Fraser University, Burnaby, BC, Canada

**Keywords:** Cancer, voltage-gated sodium channels, prostate cancer

## Abstract

Voltage gated sodium channels (VGSC) are implicated in cancer cell invasion and metastasis. However, the mechanism by which VGSC increase cell invasiveness and probability of metastasis is still unknown. In this review we outline lesser known functions of VGSC outside of action potential propagation, and the current understanding of the effects of VGSC in cancer. Finally, we discuss possible downstream effects of VGSC activation in cancer cells. After extensive review of the literature, the most likely role of VGSC in cancer is in the invadopodia, the leading edge of metastatic cancer cells. Sodium gradients are used to drive many biological processes in the body, and invadopodia may be similar. The function of the sodium hydrogen exchanger (NHE) and sodium calcium exchanger (NCX) are driven by sodium gradients. Voltage gated calcium channels, activated by membrane depolarization, are also capable of becoming activated in response to VGSC activity. Changes to hydrogen ion exchange or calcium handling have functional consequences for invadopodia and would explain the relationship between VGSC expression and invasiveness of cancer cells.

## Introduction

Voltage gated sodium channels (VGSC) are found in cancers of the breast[1], colon, lung [2], prostate [3], cervix [4], ovary [5], lymphomas [5], and melanomas [5]. VGSC are known for their function in the generation and propagation of action potentials in electrically excitable cells such as neurons and muscle fibers. However, their role in cancer remains unknown. VGSC are strongly upregulated and conserved across many different types of cancer [1,6] suggesting that they impart some advantage or survivability to cancer cells.

**Figure 1.**
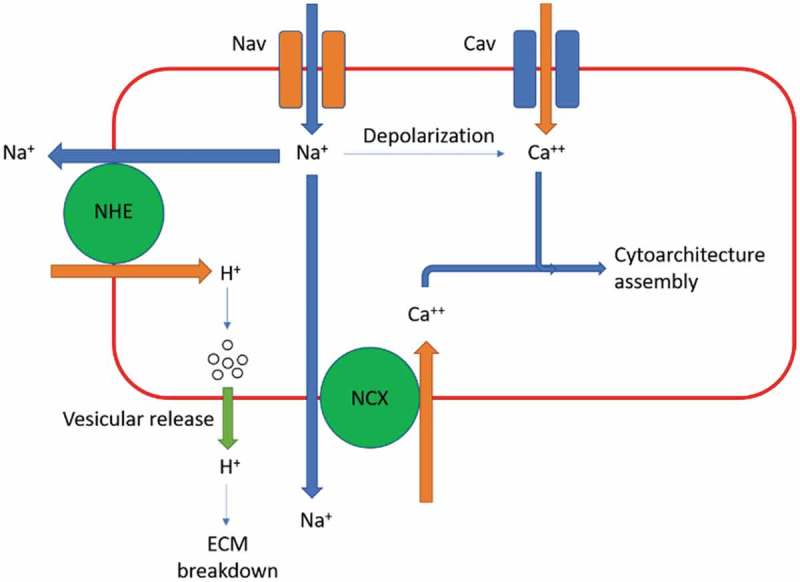
Hypothesis for mechanisms by which VGSC increase invasiveness in cancer cells. This schematic summarizes potential downstream targets of VGSC.Sodium is a driving force for many transporters and exchangers, most notably NCX and NHE. VGSC will depolarize the membrane sufficiently to activate other voltage gated ion channels such as voltage gated calcium channels. VGSC can thus affect hydrogen ion exchange and calcium handling which has functional consequences for invadopodia.

VGSC expression increases cell motility and invasiveness in cancer cells [1–6]. Although exact mechanisms in cancer cells remain unknown, VGSC are found in several non-cancerous, non excitable cell types found in microglial cells [7,8] and macrophages [9,10] where they are thought to play a role in assisting cells to degrade the extracellular matrix (ECM) and migrate rapidly through tissue microenvironments. Cancer cells may use VGSC in a similar way to assist in cell invasion. Further, under normal conditions, VGSC interact with the ECM through their β subunits [11].

Expression of VGSC has been associated with increased movement, contact independence, and metastatic potential in cancer. This review discusses the structure and function of VGSC, VGSC expression in podosomes, and implications for VGSC expression in cancer cells. Finally, we offer some ideas about what may be the specific mechanism of action through which sodium channels affect the metastatic properties of cancer cells (Figure 1). 

## Structure and function of VGSC

### Structure

Different subtypes of VGSC are differentially expressed throughout the body. Nav1.1, Nav1.2, Nav1.3, Nav1.6, Nav1.7, Nav1.8 and Nav1.9 are expressed in nervous tissue whereas Nav1.5 and Nav1.4 are expressed in skeletal and cardiac muscle respectively. The VGSC family consists of nine different α-subunits (Nav1.1–Nav1.9) and four β-subunits (Navβ1–Navβ4). Each sodium channel is made up of an α subunit forming the channel pore and two auxillary β subunits.

### The *α* subunit

The α subunit consists of four domains (I, II, III and IV) each with six transmembrane segments, S1-S6 [12]. The S1-S4 segments serve as the voltage sensor, whereas segments S5 and S6 line the inside of the channel pore [12]. The linker between domains III and IV is the fast inactivation gate, which operates in a “hinged lid” fashion to inactivate the channel shortly after opening [13].

S4 movement is responsible for channel pore opening and causes activation of the channel. In response to membrane depolarization an S4 segment from each of the four domainsmoves outward. The S4 segments contain regularly spaced arginine and lysine residues which are positively charged [14]. When the membrane becomes depolarized the intracellular milieu becomes more positive. This change in membrane potential repels the positively charged S4 segments outwards, which opens the channel.

The selectivity filter of the channel is formed by residues within segments S5 and S6. The selectivity filter is designed in such a way that it can distinguish between ions of similar size and charge. The innermost ring of the selectivity filter is made up of four residues, aspartate, glutamate, lysine and alanine, one from each domain [15]. Sodium is drawn to the negatively charged glutamate residue and is subsequently conducted through the channel. Potassium forms a weaker interaction with the glutamate than sodium, and is repelled more strongly by the positive lysine residue [15]. The size of the pore is as also a contributing factor to the higher affinity of sodium. Potassium requires a larger pore because potassium can’t shed its hydration shell. Sodium channels have smaller pore openings, so potassium is less likely to permeate. In this fashion the selectivity filter allows for a much higher affinity for sodium than other cations.

### Channel opening

There are two gates in the sodium channel, an activation gate and an inactivation gate. The activation gate is closed at rest, rapidly opens in response to depolarization, and rapidly closes in response to repolarization [16]. In the activated state the S4 segments move outwards, repelled by the positive intracellular milieu. Movement of the S4 segment causes a conformational shift in the channel that causes it to open. The S4 helix is tied to the S5 helix so when the S4 moves it tugs on the S5, which causes the channel to open [17]. When the channel is open, the pore must fill with water before it can conduct any ions. When the pore becomes filled with water sodium ions are free to be conducted into the cell.

### Fast inactivation

There are two types of inactivation, slow and fast. While fast inactivation occurs during the time frame of miliseconds, slow inactivation takes seconds, or even minutes to occur [18]. Slow inactivation takes longer to occur than fast inactivation and takes the channels longer to recover from.

The inactivation gate follows different voltage dependence and kinetics from those of activation, remaining open at rest, and closing slowly on depolarization and opening slowly in response to repolarization. Fast inactivation results in blocking the inner pore mouth with the hinged list, where shortly after depolarization the lid closes on the mouth of the pore, blocking ion permeation. The “lid” in the hinged lid model consists of three residues within the Domain III-IV linker, isoleucine, phenylalanine, and methionine (IFM). These hydrophobic residues are attracted to the hydrophobic residues in the pore exposed with the S4 movement. Fast inactivation may be removed by mutating the IFM motif and can be restored by adding free peptides containing the IFM motif [12,17]. Cleavage of the linker between domains III and IV has also been shown to greatly reduce fast inactivation [19].

### Slow inactivation

Slow inactivation is an inactivated state distinct from fast inactivation. In the fast inactivated state the pore is “plugged” by the inactivation gate. In slow inactivation, the pore itself collapses [20]. This pore collapse has a much longer recovery period than the fast inactivated state. Slow inactivation can be distinguished from fast inactivation on the basis of kinetics and pharmacology. Fast inactivation occurs over a period of milliseconds whereas slow inactivation occurs and recovers over a period of seconds to minutes [20]. During a prolonged depolarization, the S4 segments continue to move outwards, causing the pore to collapse.

### β subunits

The α subunit can be expressed alone to produce a functional channel; however, β subunits are important in membrane expression and gating kinetics [11]. Expression of β subunits increases rates of activation and inactivation [11]. β subunits have also been found to greatly increase membrane expression of the channel [11]

β subunits are made up of an extracellular N terminus which contains an immunoglobulin domain and an intracellular C-terminus [11]. The immunoglobulin domain of the β subunits is structurally similar to cell adhesion molecules (CAM’s). [11] β subunits can also function as CAMs and have been shown to play roles in cell migration, cell aggregation, and interact with the cytoskeleton [11].

## Function

VGSC conduct sodium ions into the cell when open which depolarizes the cell membrane. This feature is most commonly exhibited in the action potential, where VGSC are responsible for the generation of the upstroke of the action potential. A small depolarization, sufficient to surpass the threshold of activation, must occur in order to activate and open VGSC. In neurons, for example, excitatory and inhibitory impulses from presynaptic neurons will sum at the axon hillock of the neuron where the sum of excitatory impulses must surpass a threshold of activation in order to activate VGSC. In the absence of a depolarization sufficient to surpass the threshold VGSC will not open and action potentials, and the neuron will not fire. Once activated VGSC will open, conduct sodium ions rapidly down their concentration gradient into the cell and depolarize the cell sufficiently to activate neighboring voltage gated potassium channels responsible for the repolarization of the action potential. In this fashion VGSC are critical for the transmission of neural impulses. The general features of activation, channel opening and inactivation is common to channel function for all of the VGSC isoforms.

## Alternate functions of VGSC

VGSC are also found to a limited extent in non excitable cells like microglial cells such as astrocytes [21], oligodendrocytes [22] and schwann cells [23], and in immune cells such as macrophages [9] and dendritic cells [24]. VGSC are also found in T lymphocytes, osteoblasts, endothelial cells and fibroblasts [25]. Their functional role in these cells is not clearly established; however, they are thought to affect endosomal acidification in phagocytic cells and podosome formation in migratory immune cells [25].

Most of the functional data on VGSC activity in non excitable cells are from experiments using VGSC inhibitor tetrodotoxin (TTX). TTX administration blocks the pore of VGSC and prevents sodium ions from entering the channel, thus the resulting changes in cell function in the presence of TTX can be used to discuss likely VGSC functions. In astrocytes TTX increases Na+/K+ ATPase activity and increases rates of apoptosis [26]. In dendritic cells, TTX administration prevents cell migration [24]. TTX can also reduce insulin release from pancreatic β cells [27,28]. In macrophages, TTX reduces endosomal acidification and negatively impacts phagocytosis [9]. TTX also disrupts podosome formation and cell migration in macrophages [29]. In addition to disrupting function in both macrophages and microglia, TTX also increases local inflammation in surrounding tissue [10]. TTX also reduces phagocytic ability by over 40% in microglial cells [10]. These findings suggest that VGSC chiefly assist in endosomal acidification required for phagocytosis and podosome formation for cell invasion in immune cells. A role of VGSC in cell invasion is particularly interesting, as metastatic cancer cells are highly invasive and have the ability to migrate rapidly through tissue microenvironments.

## Podosomes: VGSC promote invasiveness in immune cells

Podosomes are structures that enable migratory immune cells to invade through tissue by forming actin rich protrusions in the leading edge of the cell and breaking down ECM components by secretion of matrix metalloproteases (MMPs) [30]. These features enable cells capable of podosome formation to invade rapidly through tissue microenvironments [30]. Cells that contain podosomes include microglial cells [7,10] in the central nervous system and macrophages [9,10] in the rest of the body. These cells contribute to the immune response by moving rapidly through tissue microenvironments to phagocytose debris and pathogens and help to rid the body of damaged cells and infectious agents. Podosomes are simple structures characterized by an actin bundle surrounded by a ring complex. The actin core is made up of actin and several actin coordinators such as Arp 2/3, WASP, and cortactin. The surrounding ring is made up of adhesion and scaffolding proteins, and is rich in integrins, viniculin and talin [7,31]. Matrix proteins degraded by secreted MMPs include fibronectin, collagen, and laminin [32]. MMPs involved in ECM degradation include serine proteases, ADAMs (a disintegrin and metalloproteinase) or matrix metalloproteinases [33,34]. Cells with podosomes have little difficulty navigating through the basal lamina and through very dense ECM with an abundance of collagen. These features enable cells containing podosomes to digest the ECM and invade rapidly through tissue.

The function of VGSC in podosomes is the most relevant to their proposed function is in cancer cells. Podosomes are VGSC dependent structures [8,9,29]. VGSC are abundant in podosomal membranes, where they are believed to have a functional role affecting cell invasiveness, [9,10,29].

## Function of VGSC in cancer

*De novo* expression of VGSC promotes cell proliferation and invasiveness in various types of cancers. Different VGSC channel isoforms are overexpressed in different forms of cancer. Nav1.5 is the predominant isoform expressed in breast, ovarian, and colon cancers, Nav1.6 in cervical and prostate cancers and Nav1.7 in lung cancers [4]. All four β subunits are also detected in cancer, β1 being expressed in the greatest quantity [4]. It appears it is not the presence of a particular VGSC that conveys malignancy, but the presence of VGSC in general that allows the cancer cells to better survive.

Expression of VGSC is linked to increased cell motility, rate of proliferation, and metastatic potential in cancer cells compared to cancer cells which do not express VGSC. In breast, prostate, and lung cancer, inhibition studies using TTX show reduced cell extension, galvanotaxis, endocytosis, migration, and cell invasion [1,2,6,35]. Recent studies also demonstrate that VGSC channels regulate angiogenesis of epithelial tissues near tumors [6]. The α subunit expression correlates to the metastatic potential of several cancers. The amount of VGSC expression can be used successfully as a marker to grade tumor severity and metastatic potential [36]. Further, among patients with the same grade of breast cancer, those with elevated expression of Nav1.5 were more likely to have a recurrence or die within five years and were more likely to develop metastasis [37]. In several studies tumor grade and cancer invasiveness correlated positively with VGSC expression [38,39]. Correlation, however, does not indicate causation. It remains unclear whether VGSC expression causes cancers to become more likely to metastasize or whether VGSC expression is simply coincidental. Mechanisms by which VGSC acts in cancer cells remain unknown.

Experiments in which VGSC channel blockers and openers were applied to cancer cells have demonstrated that VGSC functions to increase invasiveness in cancer cells. Cell motility and cell invasiveness is reduced in the presence of TTX [2,3,25,37,40]. TTX also reduces extracellular acidification in migrating cells [35]. Impacts of VGSC on cancer cell movement has been demonstrated primarily through the use of invasion chambers to measure invasiveness and scratch assays to measure cell motility. In breast cancer TTX reduces invasiveness in MDA-MB-468 cells and in MCF-7 cells [41]. In prostate cancer, TTX reduces invasiveness in LNCaP cells, and a decrease in invasion in PC3 and LNCaP cells [42].

One study examined the role of VGSC inhibition on metastasis in animal models with positive results [43]. In rat models TTX administration reduced lung metastasis in prostate cancer by > 40% and increases lifespan [43]. This study was the first *in vivo* demonstration that VGSC inhibition reduces metastasis.

## Invadopodia

VGSC are expressed in podosomes where they are thought to have a functional role in cell invasion. If cancer cells had a similar structure this would be a neat explanation of why VGSC expression increases invasiveness in cancer, and why when VGSC are inhibited with TTX cell motility, cell invasion, and metastasis is compromised. In fact, cancer cells possess a sister structure, called invadopodia, which are functionally and morphologically very similar to podosomes. Invadopodia were originally named for their ability to enable cancer cells to invade rapidly through tissue, however, some experts now think invadopodia and podosomes are the same structure entirely.[31] It appears the term invadopodia is reserved for invasive structures found in cancer cells, while the term podosomes is used for structures found in other non-cancerous cells. Due to the fact that many of the molecular markers and key functional players in podosomes are also found in invadopodia [31], it is likely that VGSC are expressed in invadopodia in the leading edge of cancer cells. More research is needed to say for certain, however, VGSC localization in invadopodia would explain why VGSC expression has effects on invasion and metastasis [2,36,43].

## How is upregulation possible?

### Epigenetic dysregulation

Cancer cells show extensive reprogramming of epigenetics including DNA methylation, histone modification, nucleosome positioning and microRNA expression [38,44,45]. Genetic changes are a widely accepted cause of carcinogenesis, where increased cell proliferation and metastatic potential are conveyed by mutations in key genes that regulate the cell cycle. It is now accepted that cancer cells have drastically different epigenetics compared to somatic cell lines. Cells undergo epigenetic changes both in the initiation of cancer as well as throughout cancer progression [46]. Genes can be silenced, upregulated, or spontaneously expressed by changing DNA methylation patterns or histone packing [38,44,45]. Therefore, it is possible that VGSC may be able to be spontaneously expressed in tissues that do not normally express VGSC due to drastic changes in epigenetic regulation of cancer cells. More research is needed to say for certain, but it is likely that spontaneous expression of the VGSC genes is the result of alterations in DNA methylation patterns and histone packing to “un-silence” the genes.

## Neuroendocrine differentiation

VGSC expression in cancer has sparked particular interest because as cancers become more aggressive the cells can take on features of neurons and endocrine cells that are not present in their tissues of origin. When a cancer cell undergoes neuroendocrine differentiation, the cell accumulates markers that are typically found in neurons. Neuroendocrine differentiation has been studied to the greatest extent in prostate cancer [39,47–49] but can also occur in other cancers. Cancers such as cervical[50], breast [51,52], thymus[53], small cell lung[54], and non-small cell lung[54] have also been shown to differentiate into neuroendocrine cells in the last stages of the disease.

It is interesting that VGSC, hugely abundant in neurons, are expressed most highly in later stages of cancers when neuroendocrine differentiation is likely to occur. It is unclear whether VGSC expression is coincidental and unrelated, or whether it VGSC expression occurs with along with that of other markers of neuroendocrine differentiation. It has not yet been examined if VGSC are present significantly higher in neuroendocrine cancer cells compared to pre-neuroendocrine differentiated cancer cells. It may be that expression of these channels is upregulated along with other neural and endocrine markers as cancer cells undergo neuroendocrine differentiation.

## Mechanistic speculations

The following discussion highlights several theories in the literature and some purely speculative ideas for why VGSC expression may be advantageous to cancer cells. An extensive review of the literature leads us to conclude that the most likely scenario is that VGSC participate in the formation of invadopodia. This is most heavily supported by their established functional role in invasion in macrophage and microglial podosomes. VGSC localization in invadopodia would explain why inhibiting VGSC causes a decrease in invasiveness and cell motility in cancer cells. However, this does not fully answer the question as to what their specific role is. Why a voltage-gated sodium channel? What are the downstream targets of sodium entry that impact invasion?

Sodium entry alone would not explain why cancer cells which express VGSC have increased survivability and invasiveness. Following sodium entry, however, there may be a downstream target that promotes invadopodia formation and function. Sodium gradients drive many energetically unfavorable physiological processes. Examples of transporters that require a sodium gradient include the sodium hydrogen exchanger (NHE) [55], the sodium calcium exchanger (NCX)[56], the Cl- anion exchanger that operates in parallel with NHE [57], and sodium glucose transporters SGLT1 and SGLT2 in the small intestine and in the nephron [58]. Of these sodium gradient driven transporters, two seem likely to have an impact in invadopodia; NCX and NHE.

NCX facilitates sodium movement down its concentration gradient and calcium movement in the opposite direction. Normally, sodium is at a higher concentration in the ECM and is transported into the cell, with calcium moved out of the cell. However, NCX can also operate in reverse-mode to bring calcium into the cell. Calcium ions are needed for many physiological processes such as vesicle transport and exocytosis [59], signal transduction where they act as a secondary messenger [59], muscle contraction [60], and is used as a cofactor in many biological reactions. A rapid influx of sodium ions through VGSC might have a downstream effect on NCX, which would alter calcium handling for events such as vesicle exocytosis or signal transduction in invadopodia.

Inward current through VGSC would also sufficiently depolarize the membrane to activate a voltage gated ion channel such as a calcium channel [61]. This can be seen in neural synapses for example, where voltage gated calcium channels are activated by depolarization near the axon terminal and bring in calcium to assist in vesicle docking and release of neurotransmitters into the synaptic cleft [61]. Although voltage gated calcium channels do not require a sodium gradient, they can be activated by membrane depolarization, which occurs near VGSC. This process can occur independently of NCX and would result in a similar increase in intracellular calcium that would alter calcium handling in the cell. In addition, recent studies have found that voltage gated calcium channels are also upregulated in later stages of cancer [49,62,63]. This suggests that upregulation of voltage gated calcium channels could be occurring in parallel with upregulation of VGSC channels. Upregulation of voltage gated calcium channels would have a similar effect on invadopodia to upregulation of NCX.

NHE is another transporter that is directly affected by sodium gradients in a cell. NHE transports sodium down its concentration gradient in exchange for H+ ions transport in the opposite direction. As with the NCX, NHE can also operate in reverse-mode. NHE is responsible for a number of things such as regulation of cell volume and pH [64], regulation of pH of lysosomes [65] and endosomes [66], and cell adhesion to the ECM [64]. Cytoskeletal anchoring of NHE also has indirect effects on migration, cell proliferation, and apoptosis [64]. NHE may play a key role in acidification to assist in ECM degradation, an important property of metastatic cells. NHE aids in acidification of vesicles, which could then be released into the ECM, or could directly acidify the ECM near the leading edge of the cell. Tumor hypoxia induces the expression of NHE in invadopodia in breast cancer cells [67]. NHE also impacts invasiveness by regulation of MMPs [67] and cathepsins [67]. In this scenario, VGSC may bring sodium in, and bring hydrogen ions out of the cell into the ECM. Although this makes sense, it is more likely that acidification of the ECM and secretion of MMP’s occurs in a more controlled and regulated fashion, such as vesicle formation.

Sodium permeation through VGSC increases cytoplasmic sodium concentrations relative to the inside of endosomes and may result in Na+ ions entering endosomes, and H+ ions being shuttled into the cytoplasm. This scenario would result in less H+ in endosomes and the cytoplasm becoming acidic, which seems unlikely. The answer must then be more complex than that. Although few studies have been conducted examining vesicle formation in cancer cell invasion, some have described vesicle formation in the context of MMP release in cell invasion [68–72] However, is not generally accepted whether ECM degradation occurs due to vesicle formation and release, or by direct acidification near the plasma membrane.

In endosomal acidification under normal conditions, sodium gradients are used to activate NHE to transport sodium into the vesicle and hydrogen out of the vesicle and into the cytosol [66]. Subsequently, an ATPase proton pump transports H+ ions down their concentration gradient into the vesicle, thus making the contents of the vesicle more acidic. A similar mechanism may occur in cancer cells, which utilize VGSC to drive the activity of NHE and subsequent ATPase proton pump activity to acidify vesicles for subsequent release into ECM. In either the case of NHE activity on the plasma membrane, or in vesicle release, it remains possible that VGSC may drive this activity.

A study by Brisson et al. is the first to add credibility to the theory that VGSC are localized in invadopodia. In this study, it was shown that Nav1.5 co-localizes with NHE-1 sodium hydrogen exchanger in invadopodia in breast cancer cells[35]. NHE-1, caveolin-1 and Nav1.5 co- immune-precipitated, which suggests that these proteins co-localize [35]. Although VGSC and NHE-1 transporters co-localize, the nature of their relationship has yet to be elucidated.

Finally, VGSC β subunits interact with both cell adhesion molecules (CAMs) and ECM proteins for cell anchoring and may also play a role in cell motility and survivability in cancer cells [11]. VGSC interactions with the ECM and CAMs may help cells detach from their original location and migrate more easily through the ECM [11]. Cells expressing VGSC are less contact-dependent than cells which express none [11]. β subunits can act as signaling molecules and interact with CAMs such as neurofascin and contactin, as well molecules found in the ECM. including tenascin [11]. VGSC β subunit interactions with CAMs and ECM proteins may allow cells expressing VGSC channels to form new interactions with the cytoskeleton and surrounding environment. As such, VGSC β subunits may assist in migration through tissue microenvironments which could be further advantageous for cancer cells

## Conclusion

In this review, we discussed (1) the structure and function of VGSC,(2) evidence for VGSC upregulation in, and increasing the invasiveness of, cancers, and (3) potential theories as to why VGSC expression increases survivability in cancer. Based on existing literature, the most likely mechanism for VGSC function in cancer is a functional role in invadopodia, with potential downstream targets such as NHE, involved in ECM degradation, or voltage-gated calcium channels or NCX effecting calcium handling in the cell. Either NCX, voltage-gated calcium channels, NHE, or all three, could be the downstream target(s) for VGSC activity in cancer cells. Finally, VGSC β subunits could play an additional role by assisting in cell adhesion in migrating cancer cells. These theories remain to be proven experimentally but may explain the functional role VGSC play in cancer metastasis.
